# Corona-Triggered Global Macroeconomic Crisis of the Early 2020s

**DOI:** 10.3390/ijerph17249404

**Published:** 2020-12-15

**Authors:** Kristijan Krstic, Ronny Westerman, Vijay Kumar Chattu, Natalia V. Ekkert, Mihajlo Jakovljevic

**Affiliations:** 1 COVID-Hospital, University Clinical Centre Kragujevac, 34000 Kragujevac, Serbia; dr.krstic.kristijan@gmail.com; 2 Federal Institute for Population Research, 65207 Wiesbaden, Germany; ronny.westerman@bib.bund.de; 3 Department of Medicine, Faculty of Medicine, University of Toronto, Toronto, ON M5G 2C4, Canada; vijay.chattu@mail.utoronto.ca; 4 Department of Public Health and Healthcare, First Moscow State Medical University (Sechenov University), 119435 Moscow, Russia; natekk@mail.ru; 5 Institute of Comparative Economics, Hosei University, Tokyo 102-8160, Japan; 6 Department of Global Health Economics and Policy, Faculty of Medical Sciences, University of Kragujevac, 34000 Kragujevac, Serbia

## 1. Corona-Triggered Global Macroeconomic Crisis of the Early 2020s

Long-lasting economic recessions spreading from initial cradle markets worldwide should be a periodic event inherent to capitalism as a prevailing socio-economic model. These crisis events unfold in relation to long-term business cycles. Their severity ranges from mild recessions to economic depression [1]. The National Bureau of Economic Research (NBER) observes the business cycle as a natural occurrence within capitalism. NBER claims that periods labelled depression “are marked by a substantial and sustained shortfall of the ability to purchase goods relative to the amount that could be produced using current resources and technology” [2].

According to credible economic history sources, such market disturbances are neither limited in frequency nor geographic extension. Recessions have occurred at least 47 times in the USA since the Article of Confederation Era was initiated in 1777 [3]. Additionally, they took place across a vast array of national jurisdictions ranging from Latin America to Greece, Finland or Far East Asian economies. Each time, with their local peculiarities and complex underlying drivers, they shaped unique historical evolution. Common market regulating mechanism failures have manifested through the rapid growth of unemployment rates, fall of real GDP, inflation and changes in consumer demand and industrial and agricultural activity. An array of local factors has driven almost every single event, but usually one prevails so much that the entire recession or depression period is nicknamed after it. Thus, we know that The Great Depression was triggered by the Wall Street stock market crash as of 4 September 1929 which has lasted until 29 October 1929 [4]. We also know that the 1973–1975 recession was directly caused by the 1973 oil crisis and the fall of the Bretton Woods system [5]. The third event that took place outside the Political or Collective West was the so-called Post-Communism [6] or Russian recession of the 1990s triggered by the preceding geopolitical events and consecutive fall of consumer demand and purchasing power in most of the socialist countries of the Warsaw Pact [7]. The fourth most notorious economic crisis of our days was, of course, the Great Recession. It commenced with the bankruptcy of Brother Lehman, reaching its peak between 2007 and 2009. It was also nicknamed the global financial crisis (GFC). Its spreading worldwide was considered to be the most severe recession since the Great Depression back in the 1930s.

Economic history records and their interpretations date back to more than two millennia of mankind’s past. The Agnus Madisson Project is a convenient example [8]. Yet the reliability of our understanding of the overall macroeconomic landscape of ancient societies and statehoods falls rapidly as we go deeper into the past beyond Colonial Age into the Middle Ages and Antiquity. This is notable to our understanding of how a single event such as a large epidemic of an infectious disease can cause a contraction of economic activity (negative real GDP growth). It is likely that the first hint would be the most notorious bubonic plague (caused by Yersinia pestis) epidemics such as the European Black Death. It spread from 1346 until approximately 1353 with attributable assessments of casualties ranging from 75 to 200 million [9]. Given the population size of the XIV century Middle East and Eurasia, it ended the previous long period of prosperity. This is probably the most notable historical example when of a pandemic causing such a tremendous halt in the overall societal level of economic activity triggered by a prolonged mortality crisis [10]. In far closer perspective, the last pandemic event of a huge scale was the Spanish flu of the early 1920s. It lasted from 1918 to 1920 and brought economic activity to a standstill as large masses of citizens worldwide became incapacitated [11].

On 31 December 2019, Chinese authorities reported to the national WHO Office an occurrence of new unidentified cases of pneumonia. For Europe, the first cases occurred also in late 2019, especially in Northern Italy, Region of Milan. Viral strains supposedly conveyed by wild animal sources were identified in the city of Wuhan, Hubei province. By 11 January 2020, China made genome sequencing of novel Coronavirus publicly available [12]. Regardless of the unconfirmed origins of this viral strain, WHO declared the COVID-19 pandemic on 11 March 2020 [13]. This has been heavily debated internationally, regarding whether it was a premature decision. Yet it took place officially, and consequences were to follow—various acts enacted by a large number of national governments accumulated over the past year. Surprisingly, although we live in an era of rapidly escalating competition among leading powerhouses, there was an unprecedented degree of unity in the global response to Coronavirus spreading. Unlike any previous time in the modern era, these interventions included border shut-down and stringent control of the flow of passengers across land, sea and flight routes among countries [14]. Even more, in the case of large or most heavily affected nations such as China, Iran, Italy, Spain, the U.S., strict control of passenger flows within single nations among large cities and regions was also assumed. The entire set of complex, mostly preventive public health measures aimed at social distancing among the citizens of the huge number of affected nations worldwide soon became known as “The Great Lockdown” [15].

Quarantine policies were first implemented with a substantial success rate in the city of Wuhan, Hubei province itself. These measures were exceptionally ambitious and infection site containment took place at a scale previously unknown in medical history [16]. Individual citizens were assigned QR-codes to track their movements in the quarantine area. Food and consumer goods purchased were even delivered by cargo drones directly to the apartment terraces of citizens with confirmed infection. Furthermore, street flying drones used face-recognition software and tracked citizens [17]. Artificial intelligence algorithms compiled all of the available inputs into the complex Big Data sets. All of these procedures brought China unseen epidemic eradication success rates by far exceeding high-income Western nations [18]. In these rich OECD societies, deployments and limitations of civil liberties were hard to achieve due to the legislative framework and resistance by the public at large [19]. Even after crossing the turning point in China, the epidemic continued to spread worldwide, gaining massive momentum in terms of incidence rates, associated morbidity and intensive care unit admissions. In many nations ranging from Iran, Mediterranean southern Europe up to North America, hospital capacities of pulmonary wards and ICU units soon became overloaded even among the richest of global cities such as NYC [20]. Consequences were a rapid build-up of brand-new field hospitals devoted to COVID-19 infected patients and the expansion of existing capacities. Among large nations, Russian Federation was among the leading ones adopting this strategy, whose mortality rates [21], given the size of the infected population, remained far below the average ones in other large, heavily affected nations [22]. An array of other measures followed to prevent further spreading of disease which ranged in many Eurasian countries from temporary social isolation in India [23] up to long-lasting Parliamentary-imposed restrictions of freedom of movement and harsh police-controlled curfew in all urban areas [24].

These epidemiological and more or less well-grounded textbook measures were extended to encircle areas of societal, economic activity far beyond initially targeted groups of citizens at risk. Large masses of cargo transportation hubs were brought to a halt, limiting not only the flux of passengers but also international trade [25]. First to suffer were all sorts of hospitality industries ranging from hotel chains and restaurants to spa and luxury travel facilities [26]. Down the chain of mutual interdependencies, such measures heavily affected bakeries, meat and dairy product facilities, commercial transportation companies, and fun and amusement industries ranging from cinemas to theatres. For the first time since WWII, eating out became a rarely seen landscape even in major urban megacities [27].

In the early spring months of March and April 2020, it was already publicly announced that a Corona-triggered economic recession was taking place in several dozens of countries and it is continuing to accelerate. Even early spring forecasts by the International Monetary Fund and major rating agencies assumed contraction of the World’s real GDP (gross domestic product) to at least −3% which exceeds the macroeconomic crisis of 2008–2009 by far [28]. Following these largely unpredictable developments, Corona triggered a stock market crash taking place from 20 February and lasting up to 7 April 2020. As time passed by, forecasts gradually became worse. The World Trade Organization (WTO) came up with its official forecast in early May that an −8.8% contraction of negative economic growth worldwide should be anticipated in 2020 [29]. Now, seven months ahead, in December 2020, this most pessimistic WTO assessment turned out to be closest to reality. The recent World Bank assessment that the full recovery of the world’s economy might not happen beyond the fiscal year 2025 [30]. Unlike any time in recent decades, alongside an array of crises, conflict and wars taking place worldwide, the Corona pandemic was the first event to trigger double G20 meetings held in a single year, with the last (virtual one) hosted by Saudi Arabia on 21 November 2020 [31]. A joint diplomatic communique [32] issued by the heads of state and national ministers of finance confirmed that COVID-19 is pushing the global economy “over the cliff” [33]. Deutsche Bank analysts in their recent, quite pessimistic forecast assess this unfolding de-globalization to be so massive and effective that they coined nickname: “Age of Disorder” for the upcoming years [34].

Since a wide range of industry and service-based sectors suffered heavily from national government-imposed measures, waives of unemployment began to happen one after another [35]. Companies were resolving the issue of lack of revenue streams by sending their employees on paid vacations, sick leave or simply firing those at the bottom of the contractual range with the poorest insurance coverage and lowest protection [36]. These most vulnerable groups were not highly skilled and educated labour forces but instead simple workers dealing with hygiene issues, transport services, hospitality and associated services. A large number of these citizens, even inclusive of those residing in the richest nations, lacked profound social benefits and support. The population share of these vulnerable families at risk of Corona-attributable catastrophic household expenditures or virtually sinking beneath the poverty line is now gradually growing to dozens of millions in large nations [37]. According to the last available release of official figures on jobless benefits, unemployment insurance demands submitted by citizens of working age in the USA exceeded 30 million in late June [38]. It continued to worsen further, and this trend is ongoing according to the latest official releases as of 25 November 2020 [39]. Generous unemployment insurance support packages were introduced by the US government in previous months [40]. Rare exemptions among the economy branches with a paradoxical growth during the Pandemic were pharmaceutical [41], medical device [42] and manufacturers [43]. IT sector inclusive of both hardware and software companies also harvested hefty profits alongside market penetration responsible for the provision of IT support [44] to the explosive need for teleconferencing and remote work and education from home [45].

Various national governments’ support to the majority-share threatened business sector and unemployed citizens range from decreasing or postponing taxpayer contributions, expanding social and unemployment insurance protection up to bold considerations of “dropping helicopter money” [46] among the most vulnerable layers of the population [47,48]. Yet, we all know the lessons inherited from vulnerabilities of capitalism throughout previous deep recessions and the historical Great Depression of the 1930s [49]. Legislative approvals of printing of excessive cash by the US Federal Reserve, European Central Bank and the Bank of Japan might hit back hard, like multiple boomerangs. The early prominent stage brings an instability of commodity prices and, possibly, a dangerous synergy creating a deflationary spiral [50]. Historically, this has taken place in circumstances where downward price reaction to an economic crisis has led to lower production, lower wages, decreased demand, and lower-still prices [51,52].

According to Borio, in many ways, the COVID-19 crisis is unique as this devastating recession has no economic roots, dances mainly along non-economic lines, and is truly global. In terms of pace, scale and scope, the policy response has been equally exceptional, eliciting an unparalleled concerted effort mixing monetary, fiscal and prudential policies [53]. A preliminary analysis conducted by Valensisi on COVID-19′s impact on global poverty in the light of IMF’s growth forecasts and showed that the pandemic will erode many of the gains recorded over the last decade in terms of poverty reduction. In addition, the Least Developed Countries, with only 14% of the global population, are set to represent the main locus of extreme poverty worldwide [54]. Ozili et al. highlighted how the COVID-19 health crisis has resulted in an economic crisis with spillovers to various industries, namely travel, hospitality, sports, financial sector, event industry, entertainment industry, health sector and education sector apart from the loss to oil-dependent countries and import-dependent countries. Their findings also emphasize that non-financial factors/non-economic factors can trigger both financial and economic meltdown in unprecedented ways, and to overcome this scenario in future, there is a great need to take human health factors as an important element of financial stability for the future stress tests of resilience [55].

From a developing country perspective, the impact of COVID-19 in Nigeria as analyzed by Ozili from the Central Bank of Nigeria highlighted five pathways through which the COVID-19 pandemic spilled over into the country. One, the COVID-19 pandemic affected borrowers’ capacity to service loans, which gave rise to NPLs that depressed banks’ earnings and eventually impaired bank soundness and stability. Second, there were oil demand shocks that were reflected in the sudden drop in the price of oil. The most noticeable and immediate spillover was the collapse in crude oil prices, which fell to as low as USD 30 per barrel from nearly USD 60 per barrel. Three, as many importers shut down their factories and closed their borders, particularly China, there were supply shocks in the global supply chain (Nigeria is an import-dependent country and as a consequence, it experienced a shortage of critical supplies such as pharmaceutical supplies, spare parts, and finished goods from China). Four, the national budget was also affected, and finally, the COVID-19 pandemic affected the Nigerian stock market. Major market indices in the stock market plunged when investors pulled out their investments into so-called safe havens like US Treasury bonds [56].

Likewise, Bhardwaj highlighted the situation in India which has the second-largest population in the world where COVID-19 has influenced the global economy in such a way that the Indian government is only concerned with saving people’s lives, whereas there is no focus on replenishing this country’s economic wealth and conditions. One of the worst times of human history is the skewed condition that has arisen due to the relocation of people from one state to another and the lack of jobs for many people [57]. Due to the impact of social isolation and recession in Russia, Grigoryev highlights the profound reduction in consumption of rich strata, unemployment and isolation has triggered the global drama of the pandemic. Huge numbers of people around the globe are facing the step back to Maslow’s pyramid: physiological needs and security [58].

Grinin’s research argues about the reconfiguration of the world-system due to the COVID-19 pandemic, geopolitical shifts and economic crisis. This reorganization consists of pulling up the political component of the modern world to the economic one as economic and financial globalization has gone much farther than political globalization. Economic globalization regresses and the political one “clears a way” to move forward and this pulling of the political component happens as a result of the beginning of a new world order [59]. COVID-19 has definitely impacted global food security through its massive recession and major disruptions in food value chains. Swinnen highlights the worst impact on poor people, women, children and migrants compared to the rich and hence there is a great need to make the food supply chains and food systems more resilient for the future [60].

Diverse support measures at various scales took place across China, India, Russia, Germany, EU and leading Emerging Markets [61,62]. A convenient case within the EU is Germany. It has followed a “Sonderweg” with financial support. In contrast to many other neighbouring countries, Germany increased additional financial support. There are two prominent examples: With about 10 billion Euros exceeding economic aid (“Außerordentliche Wirtschaftshilfe”), which includes financial support for November and December, the KFW-fast-facilities, Compensation Funds for Social and cultural events or for employers working for these branches, and interim-aids for self-employed [63]. This entails about 9.2 billion euros as compensation in health care, which includes 7.556 billion euros for hospitals, 550 million euros for intensive care beds, 308 million euros for prevention/rehabilitation, 5 million euros for Corona swab tests, 1.6 million euros for social services, and many more [64,65]. It is likely that these costs are not currently covered within the federal government budget. Consequently, after the pandemic, there will be compensation with additional taxes or budget cuts for a longer period.

After the entire economy lockdown took place in many world regions, major sea, land and flight routes and transportation chains became broken apart [66]. The revival of industrial manufacturing of a scale took place almost exclusively in China and associated ASEAN Southeast Asian nations to the levels comparable to December 2019. This was quite a surprising development, where newly unleashed expectations on “*conditionality*” of unfreezing many of less developed world regions arose [67]. This means that temporary policymakers’ tax-release measures and postponed fiscal duties are leading to the accumulation of serious debt by companies. This virtually means, for most small and medium enterprises (SMEs), crossing the line of affordability of such credits. The vast majority of such businesses fell into the trap of insolvency, since, in the absence of capital circulation and fertilization, their return on investment is insufficient to allow them a new fresh start on the post-Corona market. In most of the nations whose budgetary deficits do not allow overextension of these support policies for the economy, inclusive of high-income ones, this means that SMEs are about to declare bankruptcy in huge numbers, sinking entire sectors of the respective national economies. The post-Corona world will be far less globalized, but this newly established landscape is not sufficient to save these enterprises conducting business driven mostly by domestic demand. The aforementioned conditionality means that governments and transnational financial institutions such as the IMF, World Bank, New Development Bank (NDB, formerly “BRICS Development Bank”), The Asian Infrastructure Investment Bank (AIIB), The European Investment Bank (EIB) and the European Bank for Reconstruction and Development (EBRD) [68] might all request these companies to re-establish their business plans far more aligned with green and digitalization policies. The degree to which such political pressures would be feasible after Corona-induced recessions and even depressions in many countries remains to be seen [69].
